# Endoplasmic reticulum-targeting activatable nanophotosensitizers for hypoxia relief and enhanced photodynamic therapy†

**DOI:** 10.1039/d5sc00534e

**Published:** 2025-05-06

**Authors:** Shanchao Diao, Xiaowen He, Ying Wu, Likun Yin, Yuxin Huang, Wen Zhou, Chen Xie, Quli Fan

**Affiliations:** a State Key Laboratory of Flexible Electronics (LoFE) & Institute of Advanced Materials (IAM), Nanjing University of Posts & Telecommunications 9 Wenyuan Road Nanjing 210023 China iamwzhou@njupt.edu.cn iamcxie@njupt.edu.cn iamqlfan@njupt.edu.cn

## Abstract

Photodynamic therapy (PDT) is a promising cancer therapeutic modality. However, the specific targeting capability of conventional photosensitizers is relatively low, which significantly suppresses the efficacy of PDT. In this study, an endoplasmic reticulum (ER)-targeting nanophotosensitizer (TPPa-Y NP) was designed and prepared for enhanced PDT. TPPa-Y NPs are prepared by encapsulating an ER-targeting pheophorbide-a (TPPa) and a hypoxia inducible factor 1α (HIF-1α) inhibitor (YC-1) with a hydrogen peroxide (H_2_O_2_)-responsive amphiphilic copolymer (PEG-PMPAP). After internalization into tumor cells, TPPa-Y NPs may rapidly dissociate and release both TPPa and YC-1. TPPa can target ER, which leads to an enhancement in its fluorescence signal and PDT efficacy. On the other hand, YC-1 may effectively inhibit the overexpressed HIF-1α and alleviate tumor hypoxia, which can further enhance the PDT efficacy of TPPa. Both *in vitro* and *in vivo* studies demonstrate that TPPa-Y NPs have a better anticancer effect than the nanoparticles without YC-1 (TPPa NPs). Therefore, this study provides a smart nanophotosensitizer, which is able to target ER and alleviate hypoxia for PDT efficacy enhancement.

## Abstract 2

This work developed endoplasmic reticulum (ER)-targeting activatable nanophotosensitizers, which can selectively release ER-targeting photosensitizers and YC-1 within tumor cells for enhanced photodynamic therapy and hypoxia relief, respectively.

## Graphical abstract

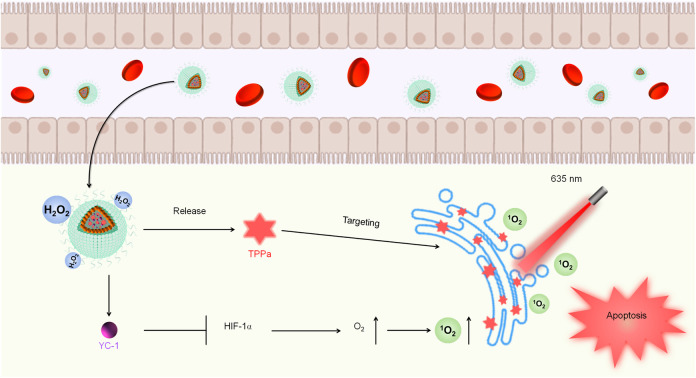

## Introduction

Photodynamic therapy (PDT) has been developed as a promising therapeutic modality against a variety of cancers.^1^ The procedure of PDT involves the excitation of photosensitizers with light and the transfer of excited energy or electrons into oxygen or biomolecules to generate highly toxic reactive oxygen species (ROS) or radicals for cancer therapy.^2,3^ The key component for PDT is a photosensitizer, whose structure determines the efficiency of ROS generation and excitation wavelength.^4,5^ Photosensitizers can be roughly divided into organic small-molecular, macromolecular and inorganic nanoparticular photosensitizers.^6^ Among them, small-molecular photosensitizers, such as porphyrin, cyanine, and boron-dipyrromethene (BODIPY), garnered attention due to their high ROS generation and biosafety, and some of them have been approved for clinical application.^7–11^ However, most small-molecular photosensitizers are hydrophobic, which causes them to aggregate under physiological conditions and greatly quenches their photodynamic effect.^12–14^

As different subcellular organelles have different functions, organelle targeting has shown great potential in cancer theranostics, which can greatly improve the specificity of treatment.^15,16^ Among all organelles, the endoplasmic reticulum (ER) is the largest organelle in eukaryotic cells and has been demonstrated to play an important role in cells. ER is involved in multiple metabolic processes, such as signaling functions, sensing and biosynthesis.^17,18^ In addition, ER is the most important organelle for protein folding, synthesis and modification, ensuring the proteomic function of cells.^19^ It has been proved that damage to ER function may lead to accumulation of unfolded proteins, causing further ER stress, and ER stress can directly cause tumor cell death.^20,21^ Recently, several studies have shown that PDT can cause severe ER stress, indicating that targeting ER is an effective approach for enhancing PDT efficacy.^22–26^

Although PDT has shown great potential in cancer therapy, the hypoxic environment of a solid tumor significantly suppresses its efficacy, as oxygen is one component in PDT.^27,28^ A variety of Type I photosensitizers, which have low reliance on oxygen, have been developed to overcome this problem.^29^ However, the design principles of Type I photosensitizers are still being explored.^30–32^ Within a solid tumor, hypoxia inducible factor 1α (HIF-1α) is overexpressed due to hypoxia.^33^ In addition, the PDT process will rapidly consume oxygen, aggravating tumor hypoxia, leading to the further upregulation of HIF-1α.^34^ The overexpressed HIF-1α may cause cancer metastasis and significantly reduce the efficacy of PDT.^35^ Studies have demonstrated that inhibiting the expression of HIF-1α can suppress metastasis and alleviate tumor hypoxia, thus improving the efficacy of PDT.^36^ Such a strategy has shown great potential in cancer therapy.

**Scheme 1:**
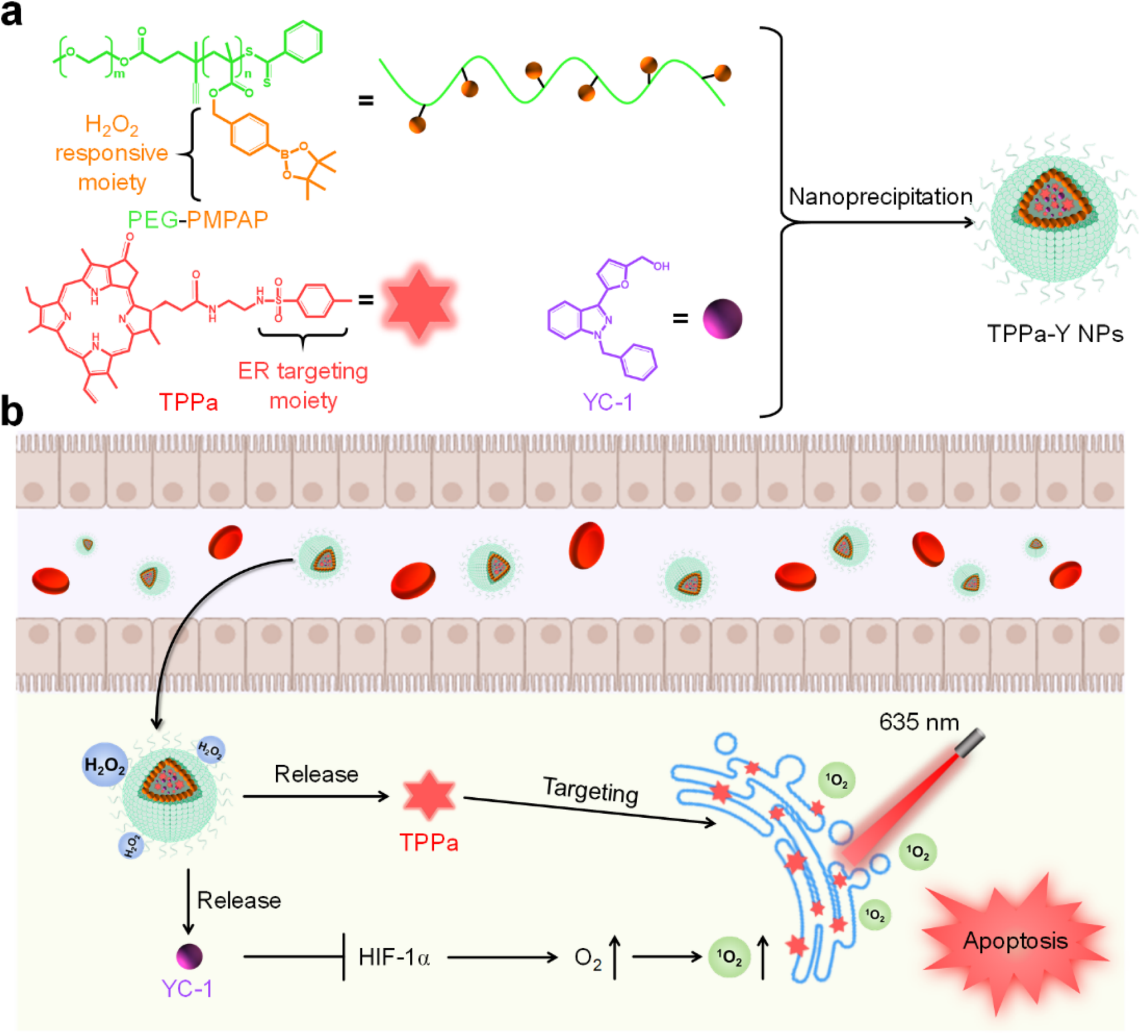
(a) Schematic illustration of the preparation of TPPa-Y NPs. (b) The process of TPPa-Y NP-mediated hypoxia alleviation and enhanced PDT.

In this study, we designed an ER-targeting H_2_O_2_-activatable nanophotosensitizer (TPPa-Y NP) for hypoxia alleviation and enhancing PDT efficacy. TPPa-Y NPs are prepared by using an amphiphilic H_2_O_2_-responsive copolymer PEG-PMPAP to encapsulate the ER-targeting photosensitizer TPPa and HIF-1α inhibitor YC-1 *via* nanoprecipitation (Scheme 1a). Upon treatment with H_2_O_2_, the phenylboronic ester moiety of PEG-PMPAP would be cleaved, making the hydrophobic part of PEG-PMPAP hydrophilic, leading to the rapid release of TPPa and YC-1. After internalization into tumor cells, TPPa-Y NPs may simultaneously release TPPa and YC-1. Owing to the ER-targeting moiety, the released TPPa could target ER, and both its fluorescence signal and photodynamic efficiency would be improved. Conversely, YC-1 may inhibit overexpressed HIF-1α and alleviate hypoxia, enhancing PDT efficacy.^37^ Thus, TPPa-Y NPs are smart nanophotosensitizers for fluorescence imaging-guided enhanced PDT.

## Results and discussion

### Synthesis, preparation and characterization of TPPa-Y NPs

To prepare TPPa-Y NPs, PEG-PMPAP and TPPa were synthesized first (Scheme S1†). PEG-PMPAP was synthesized *via* reversible addition fragmentation chain transfer (RAFT) polymerization. (4-(4,4,5,5-Tetramethyl-1,3,2-dioxaborolan-2-yl)phenyl)methanol was reacted with methacryloyl chloride to give compound 1 as a monomer with an H_2_O_2_-responsive phenylboronic ester. Then, PEG-RAFT (*M*_n_ = 10 000 g mol^−1^) was used as the chain transfer agent (CTA) for RAFT polymerization of compound 1 under the initiation of 2,2′-azobis(2-methylpropionitrile) (AIBN) to give PEG-PMPAP. The proton nuclear magnetic resonance (^1^H NMR) spectra indicated the successful synthesis of compound 1 and PEG-PMPAP (Fig. S1 and S2†). For the spectrum of PEG-PMPAP, the resonance peaks from PEG and phenylboronic ester could all be observed. The degree of polymerization of PEG-PMPAP was estimated as 11 by calculating the ratio of peak integration at 3.55 ppm and 7.32 ppm, which were ascribed to the peaks of PEG and phenyl moieties, respectively (Fig. S2†). TPPa was synthesized by reacting commercially available pheophorbide-a (PPa) with a widely used ER-targeting moiety *N*-(2-aminoethyl)-4-methyl-benzenesulfonamide *via* an amidation reaction. Both ^1^H NMR and matrix-assisted laser desorption ionization-time of flight (MALDI-TOF) mass spectrometry demonstrated the synthesis of TPPa (Fig. S3 and S4†). Both TPPa and PPa had similar absorption and emission spectra, showing that the modification had no influence on the optical properties of PPa (Fig. S5†). These results indicated the successful synthesis of PEG-PMPAP and TPPa.

**Fig. 1:**
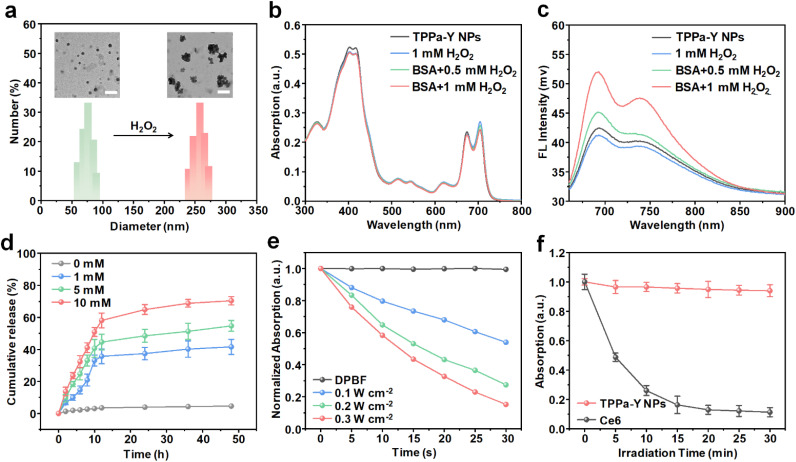
Characterization of TPPa-Y NPs. (a) Hydrodynamic size distribution and TEM image (inset) of TPPa-Y NPs before and after treatment with H_2_O_2_. The scale bars represent 300 nm. Absorption (b) and emission (c) spectra of TPPa-Y NPs under different treatments. (d) Release profile of YC-1 under different concentrations of H_2_O_2_. (e) Normalized absorption changes of DPBF incubated with TPPa-Y NPs under 635 nm laser irradiation with different powers. (f) Normalized absorption changes of TPPa-Y NPs and Ce6 under 635 nm laser irradiation over time. The error bars represent standard deviations of three separate measurements.

TPPa-Y NPs were then prepared *via* nanoprecipitation using PEG-PMPAP, TPPa, and YC-1. Dynamic light scattering (DLS) results indicated that the hydrodynamic size of TPPa-Y NPs was mainly distributed in the range of 60–85 nm. Transmission electron microscopy (TEM) images showed that TPPa-Y NPs had a spherical morphology with a diameter of around 50–70 nm (Fig. 1a). As the TEM images showed the nanoparticles in a dry state, the nanoparticle size estimated from the TEM images was smaller than the DLS result. TPPa-Y NPs showed good stability in either PBS or FBS environments, and their average hydrodynamic size remained almost the same even after storage for 28 days (Fig. S6†). The zeta potential of the TPPa-Y NPs was determined as −16.5 ± 1.58 mV, and the value remained almost the same after a 7 day-storage, confirming the good stability of TPPa-Y NPs (Fig. S7†). In contrast, the hydrodynamic size of the TPPa-Y NPs increased significantly upon treatment with H_2_O_2_, which could be attributed to the cleavage of the phenylboronic ester moiety and the aggregation of TPPa and YC-1 after nanoparticle dissociation (Fig. 1a). TPPa-Y NPs showed intense absorption in the range 650–750 nm. Compared with the absorption of TPPa in THF, the absorption of TPPa-Y NPs exhibited an obvious red-shift, indicating that TPPa may form J-aggregates within nanoparticles (Fig. 1b). In addition, after treatment with H_2_O_2_, the absorption spectrum was further red-shifted, which could be ascribed to the enhanced aggregation of TPPa after treatment. However, the red-shift was diminished when bovine serum albumin (BSA) was added with H_2_O_2_. This phenomenon indicated that BSA could stabilize the released TPPa and prevent its aggregation. The fluorescence of TPPa-Y NPs was in the near-infrared (NIR) region and the maximum emission was nearly 700 nm. After adding H_2_O_2_, the fluorescence intensity decreased slightly, showing that the fluorescence signal would be quenched after aggregation. In contrast, such intensity increased 2-fold with the addition of BSA, indicating that the dispersion of TPPa may enhance its fluorescence intensity (Fig. 1c).

The drug release profile of TPPa-Y NPs was studied by high-performance liquid chromatography (HPLC). Without H_2_O_2_ treatment, almost no YC-1 could be released after 48 h. With the increase in H_2_O_2_ concentration, the release percentage of YC-1 gradually increased in an obvious H_2_O_2_-dependent manner. Under 10 mM H_2_O_2_, up to 70% of YC-1 was released at the time point of 48 h (Fig. 1d). Under 635 nm laser irradiation, the absorption of TPPa-Y NP-incubated 1,3-diphenylisobenzofuran (DPBF) decreased rapidly, with the rate of decrease increasing with an increase in laser power (Fig. 1e and S8†). This result showed that TPPa-Y NPs had satisfactory ability for generating ROS under laser irradiation. To study the ROS species further, SOSG and DHR-123 were used as indicators. The results showed that the main ROS species generated from the TPPa-Y NPs was singlet oxygen (^1^O_2_) (Fig. S9†). In addition, the absorption of TPPa-Y NPs exhibited almost no decrease under continuous 635 nm laser irradiation for 30 min, while the absorption of chlorin e6 (Ce6) decreased by 90% after the same irradiation, indicating the excellent photostability of TPPa-Y NPs (Fig. 1f). TPPa-Y NPs also showed high photostability upon treatment with H_2_O_2_ and BSA, indicating that TPPa was able to conduct long-term PDT in a dispersed state (Fig. S10†).

### *In vitro* cellular studies

**Fig. 2:**
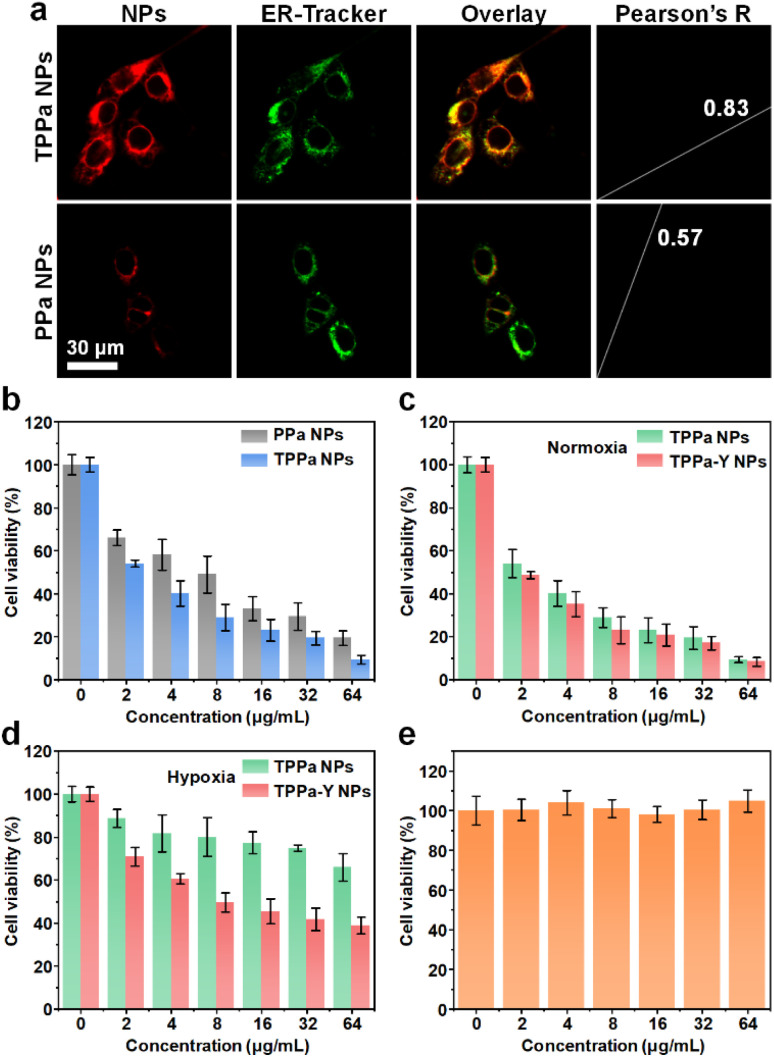
Cell studies. (a) Confocal fluorescence images of PPa NPs or TPPa NP-treated 4T1 cells. The red fluorescence signal indicates the location of PPa, while the green fluorescence signal indicates the location of ER-tracker green. (b) Viability of 4T1 cells treated with different concentrations of PPa NPs or TPPa NPs under 635 nm laser irradiation. Viability of 4T1 cells treated with different concentrations of TPPa NPs or TPPa-Y NPs under normoxia (c) or hypoxia (d) condition in the presence of 635 nm laser irradiation. (e) Viability of NIH-3T3 cells treated with different concentrations of TPPa-Y NPs. The error bars represent standard deviations of three separate measurements.

After confirming the capability of drug release and ^1^O_2_ generation, the behavior of nanoparticles within cells was studied. The ER-targeting ability of TPPa was first evaluated by confocal fluorescence imaging. Two kinds of nanoparticles, TPPa NPs and PPa NPs, were prepared using PEG-PMPAP to encapsulate TPPa and PPa, respectively. After incubation with TPPa NPs for 3 h, 4T1 cells exhibited an obvious red fluorescence signal, and such a signal was highly consistent with the green fluorescence signal from ER-tracker green, which indicated the location of ER (Pearson's *R* value 0.83). In contrast, the PPa NP-incubated cells showed a much weaker red fluorescence signal and a lower Pearson's *R* value (0.57) (Fig. 2a).

This phenomenon indicated that TPPa NPs could be internalized into 4T1 cells and that TPPa may target ER. PPa NPs may also be internalized into cells, but showed no obvious ER-targeting capability. In addition, the much higher red fluorescence signal observed in TPPa NP-incubated cells compared to PPa NP-incubated cells indicates that targeting ER could greatly enhance the fluorescence signal of TPPa. Based on previous literature, the benzenesulfonamide moiety could target an ER-overexpressed sulfonamide receptor.^38,39^ Such targeting caused the aggregated TPPa to be effectively dispersed, enhancing the fluorescence of TPPa. Under 635 nm laser irradiation, both TPPa NPs and PPa NsP could kill 4T1 cells. However, the cytotoxicity of TPPa NPs was higher than that of PPa NPs at each concentration. The cell viability under the highest concentration (64 μg mL^−1^) for TPPa NPs was much lower than for PPa NPs (10% *vs.* 20%), showing that TPPa NPs had better *in vitro* PDT efficacy than PPa NPs (Fig. 2b).

As TPPa NPs had better anticancer effect than PPa NPs, the effect of YC-1 on cellular behavior was further studied. TPPa-Y NPs were able to be internalized into 4T1 cells, as confirmed by flow cytometry analysis (Fig. S11†). Under normoxia, TPPa-Y NPs had cytotoxicity against 4T1 cells similar to TPPa NPs under 635 nm laser irradiation indicating that YC-1 had no obvious influence on cytotoxicity under these conditions (Fig. 2c). Under hypoxia, the cytotoxicity of TPPa NPs with laser irradiation was much lower than that under normoxic conditions, and the viability of the cells was even higher than 60% at a concentration of 64 μg mL^−1^. In contrast, TPPa-Y NPs still showed certain cytotoxicity under hypoxia, and the cell viability dropped to less than 40% at a concentration of 64 μg mL^−1^ (Fig. 2d). The live/dead assay and apoptosis flow cytometry analysis also confirmed these results (Fig. S12†). It was reasonable that the PDT efficacy was less effective under hypoxia than that under normoxia. The reason for the better PDT efficacy for TPPa-Y NPs than for TPPa NPs could be attributed to the inhibition of HIF-1α and alleviation of hypoxia by YC-1. Without laser irradiation, the viability of TPPa-Y NP-incubated NIH-3T3 cells was almost 100% at all concentrations, indicating the good cytocompatibility of TPPa-Y NPs (Fig. 2e).

**Fig. 3:**
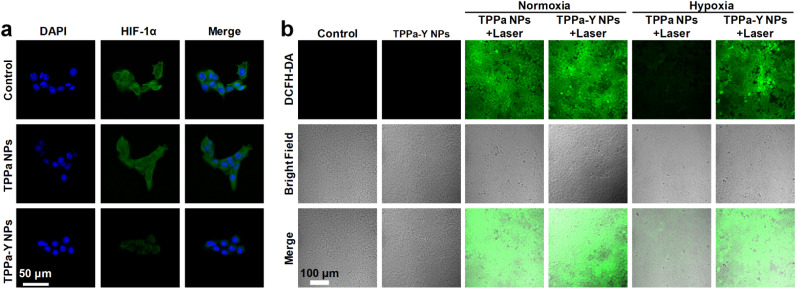
(a) Confocal fluorescence images of 4T1 cells stained with DAPI (blue) and HIF-1α antibody (green) under different treatments. (b) Confocal fluorescence images of DCFH-DA-incubated 4T1 cells under different treatments.

To study the mechanism of the superior *in vitro* anticancer efficacy of TPPa-Y NPs, the HIF-1α expression and ROS level within the cells were estimated by confocal fluorescence imaging. After incubation with TPPa NPs or TPPa-Y NPs, 4T1 cells were treated with HIF-1α antibodies to label HIF-1α within cells. For the 4T1 cells incubated with TPPa NPs, obvious green fluorescence was observed within the cells, and the intensity was similar to that of the control group. In contrast, TPPa-Y NP-incubated cells had a much lower intensity of green fluorescence than control cells (Fig. 3a). Such results demonstrated that TPPa-Y NPs are able to significantly suppress the expression of HIF-1α within 4T1 cells, while TPPa NPs may not, probably due to the encapsulated YC-1 within TPPa-Y NPs. A ROS indicator, 2′-7′-dichlorodihydrofluorescein diacetate (DCFH-DA), was used to detect the intracellular ROS level. For the TPPa-Y NP-incubated cells without laser irradiation, almost no green fluorescence signal was detected, indicating low ROS levels within 4T1 cells. Under normoxia, both TPPa NPs and TPPa-Y NP-incubated cells showed an obvious green fluorescence signal, and their intensities were almost the same. In contrast, the green fluorescence signal within TPPa NP-incubated cells was much weaker than that of TPPa-Y NP-incubated cells under hypoxia after irradiation (Fig. 3b). Such a phenomenon showed that TPPa NPs could generate ROS only under normoxia, while TPPa-Y NPs may also retain their ROS generating-ability under hypoxia, which could be ascribed to the inhibition of HIF-1α expression and hypoxia relief by YC-1 within TPPa-Y NPs.

### *In vivo* tumor imaging and anti-tumor effects

**Fig. 4:**
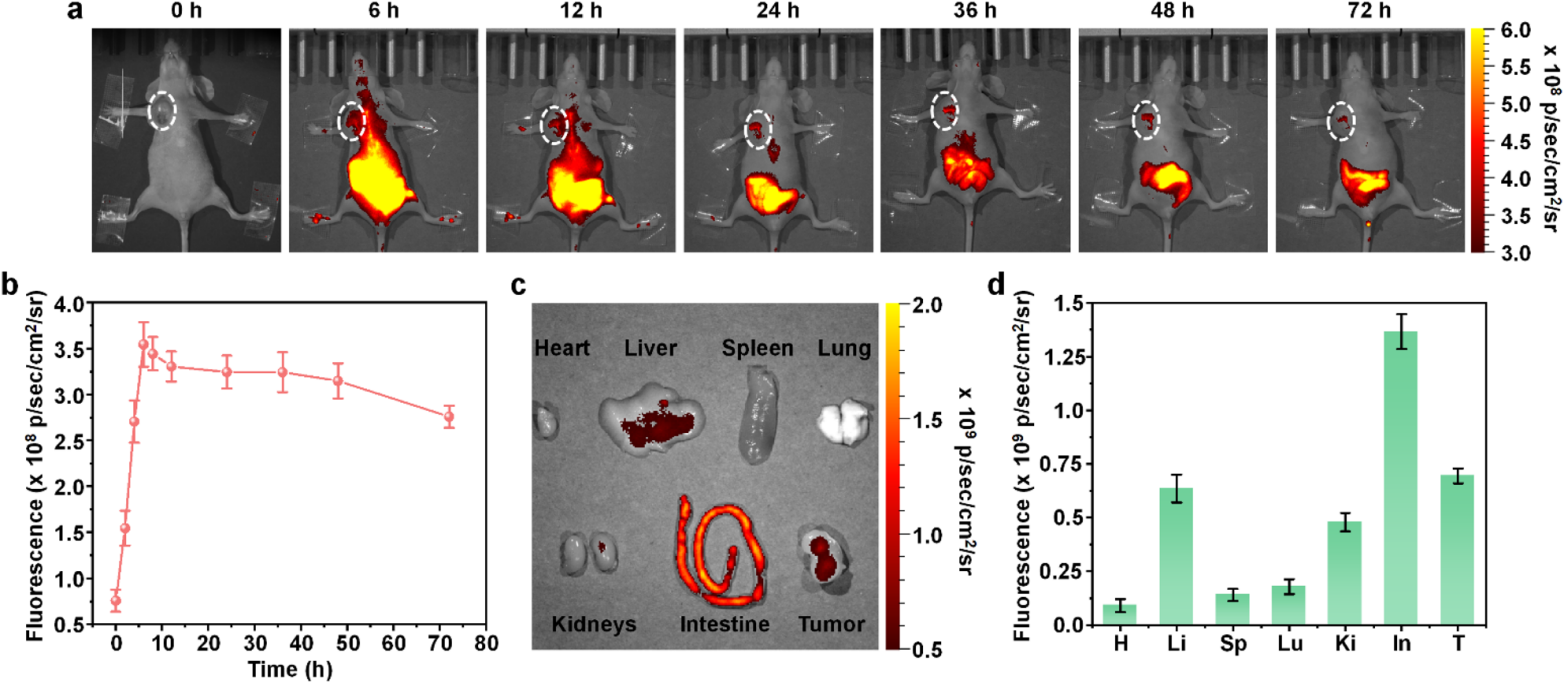
*In vivo* tumor imaging. (a) *In vivo* fluorescence images of 4T1 tumor bearing mice i.v. injected with TPPa-Y NPs at different time points. (b) Fluorescence intensities of the tumor region as a function of post-injection time. *Ex vivo* fluorescence images (c) and intensities (d) of major organs including tumor. H: heart, Li: liver, Sp: spleen, Lu: lung, Ki: kidney, In: intestine, T: tumor. The error bars represent standard deviations of three separate measurements (*n* = 3).

After confirming the *in vitro* anticancer effect of TPPa-Y NPs, TPPa-Y NPs were applied for *in vivo* fluorescence imaging. The mice were inoculated with 4T1 cells to establish a tumor-bearing mouse model. Before i.v. injection of TPPa-Y NPs, the mice showed almost no fluorescence signal, indicating the low background signal of NIR fluorescence imaging. After i.v. injection of TPPa-Y NPs, the fluorescence signal gradually increased in the liver and abdomen region of mice, indicating that TPPa-Y NPs were metabolized mainly *via* a hepatobiliary metabolic pathway. After 6 h of injection, the tumor region displayed an obvious fluorescence signal, showing that TPPa-Y NPs may accumulate into the tumor and had a good imaging effect (Fig. 4a). Furthermore, 24 h after injection, the fluorescence signal in the liver region decreased rapidly, indicating that TPPa-Y NPs undergo a relatively fast metabolic process. The rapid clearance of TPPa-Y NPs from the liver meant the tumor had a high signal-to-background ratio. The quantitative result indicated that the highest fluorescence intensity in the tumor region was detected at *t* = 6 h after injection. At such time, the fluorescence intensity of the tumor was 4.8-fold higher than that observed before injection. At *t* = 72 h after injection, the tumor fluorescence intensity was still 75% of the maximum intensity, demonstrating that TPPa-Y NPs had long-term tumor retention (Fig. 4b). After injection for 72 h, the mice were sacrificed, and the major organs were collected for fluorescence imaging. The intestine had the highest fluorescence intensity among all the organs (Fig. 4c). This phenomenon was also found in our previous PPa-related works,^40,41^ which showed that TPPa-Y NPs could be rapidly cleared out of the body *via* hepatobiliary metabolism. Besides the intestine, the tumor had the highest fluorescence intensity, and its intensity was even higher than that of the liver, proving the good tumor targeting capability of TPPa-Y NPs (Fig. 4d). These results demonstrated that TPPa-Y NPs could effectively accumulate into the tumor and NIR fluorescence tumor imaging could be carried out.

**Fig. 5:**
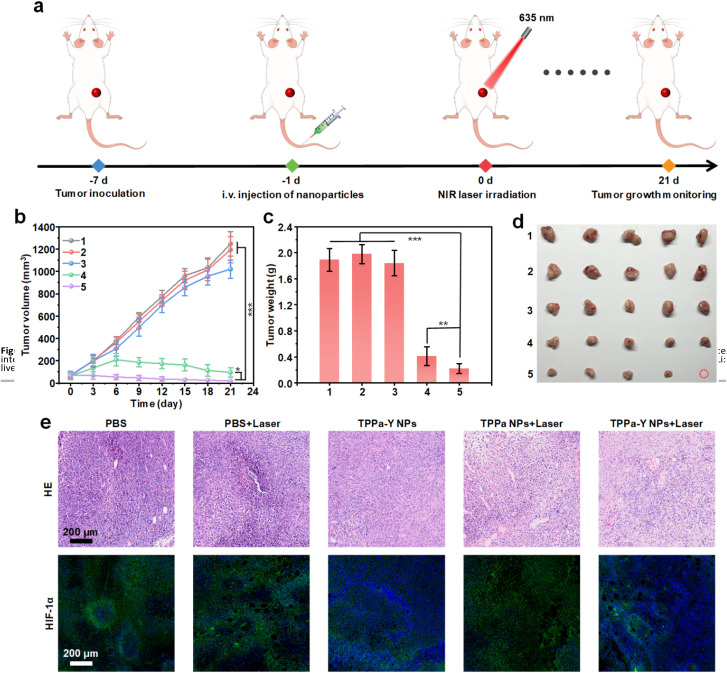
*In vivo* anticancer study. (a) Schematic illustration of *in vivo* PDT process. (b) Tumor volume of mice under different treatments as a function of treatment time. Tumor weight (c) and images (d) of mice after treatment for 21 days. (e) H&E staining and immunofluorescence imaging of HIF-1α for tumor tissue collected from mice under different treatments. 1: PBS, 2: PBS + Laser, 3: TPPa-Y NPs, 4: TPPa NPs + Laser, 5: TPPa-Y NPs + Laser. The error bars represent standard deviations of five separate measurements (*n* = 5). *p < 0.05, **p < 0.01, ***p < 0.001.

The *in vivo* anticancer efficacy of TPPa-Y NPs was then evaluated. Mice were inoculated with 4T1 cells, and after 6 days, the tumor volume reached 50–100 mm^3^. The mice were randomly divided into five groups with five mice in each group, and mice in each group received different treatments. For the groups with 635 nm laser irradiation, the irradiation was conducted once 24 h after injection. The tumor growth was monitored until day 21 (Fig. 5a). For PBS and PBS + Laser groups, the tumor volume rapidly increased. The tumor growth of the TPPa-Y NPs group was also fast, showing no significant difference from the PBS or PBS + Laser group, indicating that TPPa-Y NPs without laser irradiation had almost no tumor inhibition capability. For the TPPa NPs group, the tumor growth was greatly inhibited, and the inhibition rate could reach 86.6%, demonstrating the efficacy of PDT mediated by TPPa NPs. Among all the groups, TPPa-Y NPs + Laser showed the highest tumor inhibition rate (96.3%) (Fig. 5b). The higher inhibition rate for TPPa-Y NPs + Laser than TPPa NPs + Laser was attributed to the loaded YC-1, which may enhance PDT efficacy. At *t* = 21 days, the mice were sacrificed, and the tumors from each mouse were collected. Tumors from TPPa-Y NPs + Laser groups had the lowest weight and smallest volume among all the groups, further confirming the best anticancer efficacy of TPPa-Y NP-mediated PDT (Fig. 5c and d). During treatment, the body weight of mice in each group remained steady, indicating that none of the treatments had obvious side effects for mice (Fig. S13†).

The *in vivo* anticancer efficacy and biosafety of the treatments were then studied at the cellular level. Hematoxylin and eosin (H&E) staining of tumor tissues from each group indicated that both TPPa NPs + Laser and TPPa-Y NPs + Laser groups had a large amount of dead tumor cells, while the other three groups did not show such effects. This phenomenon demonstrated the efficacy of TPPa NPs and TPPa-Y NP-mediated PDT. Immunofluorescence staining was conducted to evaluate the HIF-1α expression level in the tumor tissue. The tumor tissues collected from PBS, PBS + Laser, and TPPa NPs + Laser groups showed a strong green fluorescence signal, indicating a high HIF-1α level in the tissue. In contrast, the green fluorescence signal of tissues from both TPPa-Y NPs and TPPa-Y NPs + Laser groups were much weaker. These results confirmed that TPPa-Y NPs could effectively suppress the expression of HIF-1α in the tumor, thus achieving better *in vivo* PDT efficacy for TPPa-Y NPs (Fig. 5e). After treatment, the major organs of mice from each group were collected for H&E staining, and the results showed that none of the treatments caused obvious damage to the organs of mice (Fig. S14†). In addition, biochemical analysis of blood from TPPa-Y NP-injected mice confirmed the good *in vivo* biosafety of TPPa-Y NPs (Fig. S15†). These results proved that TPPa-Y NPs had superior *in vivo* PDT efficacy and high biosafety.

## Conclusions

In summary, we designed an ER-targeting nanophotosensitizer (TPPa-Y NP) for hypoxia relief and enhanced PDT. TPPa-Y NPs were composed of a benzenesulfonamide-modified photosensitizer (TPPa), an HIF-1α inhibitor, and an H_2_O_2_-responsive amphiphilic copolymer (PEG-PMPAP). TPPa-Y NPs had uniform size and stable nanostructure, and showed satisfactory ^1^O_2_ generation yield with high photostability. Upon the addition of H_2_O_2_, TPPa-Y NPs may rapidly release encapsulated TPPa and YC-1, and the release percentage of YC-1 could reach 70% within 48 h. TPPa-Y NPs may be internalized into 4T1 cells, and the released TPPa could effectively target ER with the turning-on of its fluorescence signal. In addition, the targeting of ER could enhance the PDT efficacy of the nanophotosensitizer. Due to the presence of YC-1, TPPa-Y NPs demonstrated effective PDT efficacy and HIF-1α inhibition rate under hypoxia, while TPPa NPs showed no such effect. TPPa-Y NPs were able to accumulate into the tumor site *via* passive targeting and light the tumor up by NIR fluorescence imaging. Owing to the PDT and HIF-1α inhibiting effect, TPPa-Y NPs exhibited a high *in vivo* tumor growth inhibition rate (96.3%), and could greatly inhibit the expression of HIF-1α within a solid tumor.

Overall, our study designed an ER-targeting activatable nanoplatform for enhanced PDT. By using a photosensitizer with longer absorption and emission wavelengths, ER-targeting nanophotosensitizers with the capability of deep tumor theranostics could be developed. Furthermore, other types of stimuli-responsive nanophotosensitizers could also be designed based on the formulation of TPPa-Y NPs to achieve better anticancer efficacy. Based on previous literature, photosensitizers with ER-targeting ability could improve the efficiency of immunogenic cell death for immunotherapy.^42,43^ Thus, the ER-targeting activatable nanoplatform may also be utilized for photoimmunotherapy in our future work.

## Author contributions

Shanchao Diao: conceptualization, methodology, writing – original draft. Xiaowen He: conceptualization, data curation, formal analysis. Ying Wu: investigation. Likun Yin: investigation. Yuxin Huang: investigation, validation. Wen Zhou: writing – review & editing. Chen Xie: conceptualization, supervision. Quli Fan: supervision, methodology.

## Conflicts of interest

There are no conflicts to declare.

## Supplementary Material

SC-016-D5SC00534E-s001

## Data Availability

The data supporting this article have been included as part of the ESI.†
