# Supplementary material for: Endoplasmic reticulum-targeting activatable nanophotosensitizers for hypoxia relief and enhanced photodynamic therapy
Source: Chem Sci. 2025 May 6;16(24):10909–17. doi: 10.1039/d5sc00534e (PMC12086584; doi:10.1039/d5sc00534e)
Supplement: SC-016-D5SC00534E-s001 [file SC-016-D5SC00534E-s001.pdf]

Supplementary Information (SI) for Chemical Science.
This journal is © The Royal Society of Chemistry 2025

Supporting Information

Endoplasmic reticulum-targeting activatable nanophotosensitizers for hypoxia relief and enhanced photodynamic therapy

Shanchao Diao⊥, Xiaowen He⊥, Ying Wu, Likun Yin, Yuxin Huang, Wen Zhou, Chen Xie*, and Quli Fan**

State Key Laboratory of Flexible Electronics (LoFE) & Institute of Advanced Materials (IAM), Nanjing University of Posts & Telecommunications, 9 Wenyuan Road, Nanjing 210023, China

Email: iamwzhou@njupt.edu.cn; iamcxie@njupt.edu.cn; iamqlfan@njupt.edu.cn

⊥ These authors contributed equally.

1. Supporting Figures

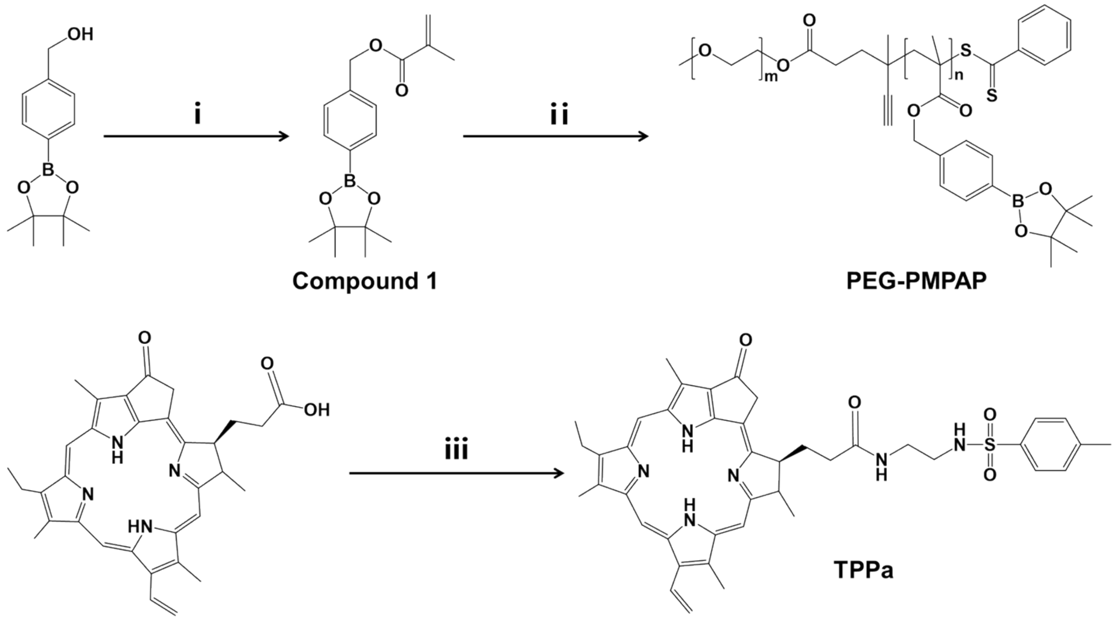

Scheme S1. Synthetic routes of PEG-PMPAP and TPPa. Reagents and conditions: (i)(4-(4,4,5,5-Tetramethyl-1,3,2-dioxaborolan-2-yl)phenyl)methanol, methacryloyl chlori de, 4-dimethylaminopyridine (DMAP), dichloromethane, room temperature (RT), 24 h; (ii) PEG-RAFT, 2,2′-azobis(2-methylpropionitrile) (AIBN), anhydrous tetrahydrof uran, N_2, 70°C, 24; (iii) pheophorbide-a (PPa), N-(2-aminoethyl)-4-methyl-benzenesu lfonamide, 2-(7-azabenzotriazol-1-yl)-N, N, N', N'-tetramethyluronium hexafluoropho sphate (HATU), 1-hydroxy benzotriazole (HOBT), N, N-diisopropylethylamine (DIPE A), anhydrous N, N-dimethylformamide (DMF), RT, 24 h.

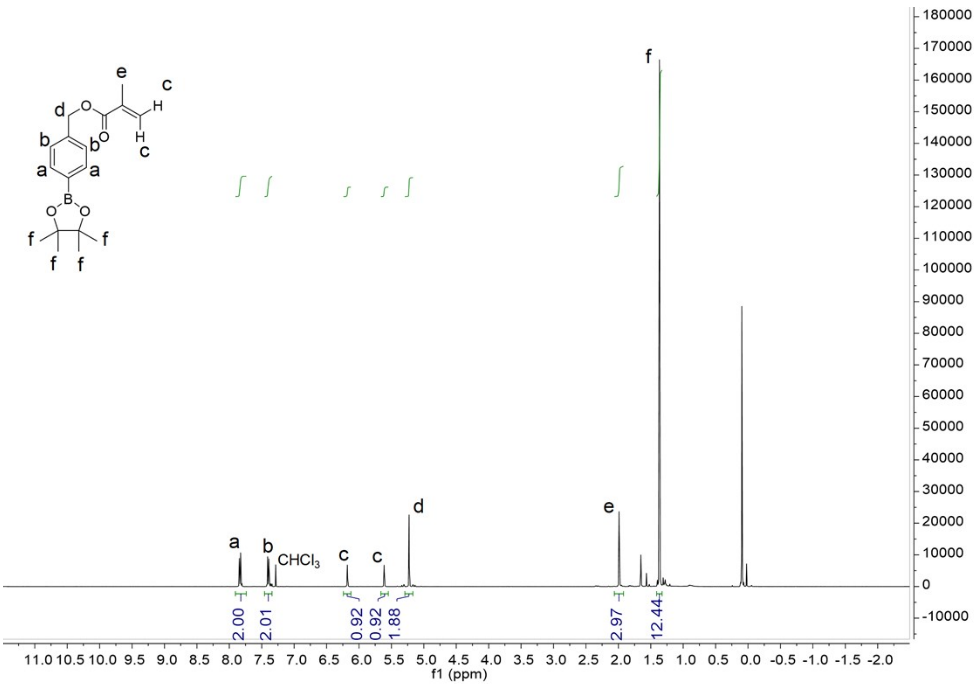

Figure S1. ^{1}H NMR spectrum of Compound 1. $CDCl_3$ was used as solvent.

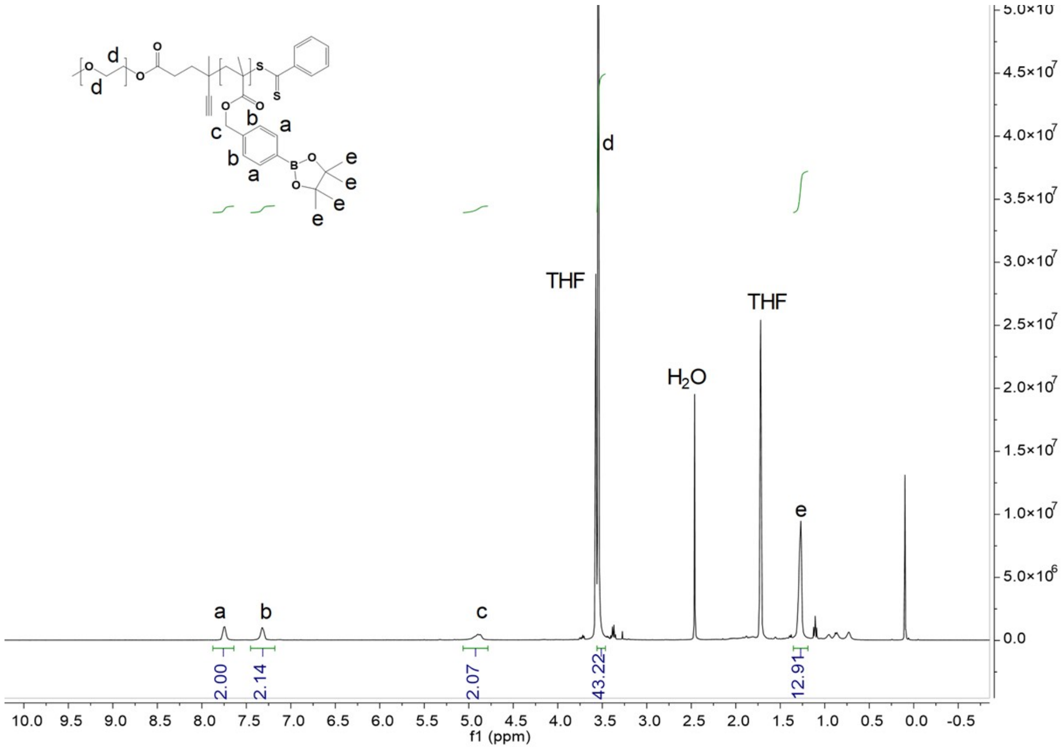

Figure S2. ^{1}H NMR spectrum of PEG-PMPAP. Tetrahydrofuran-d8 was used as solvent.

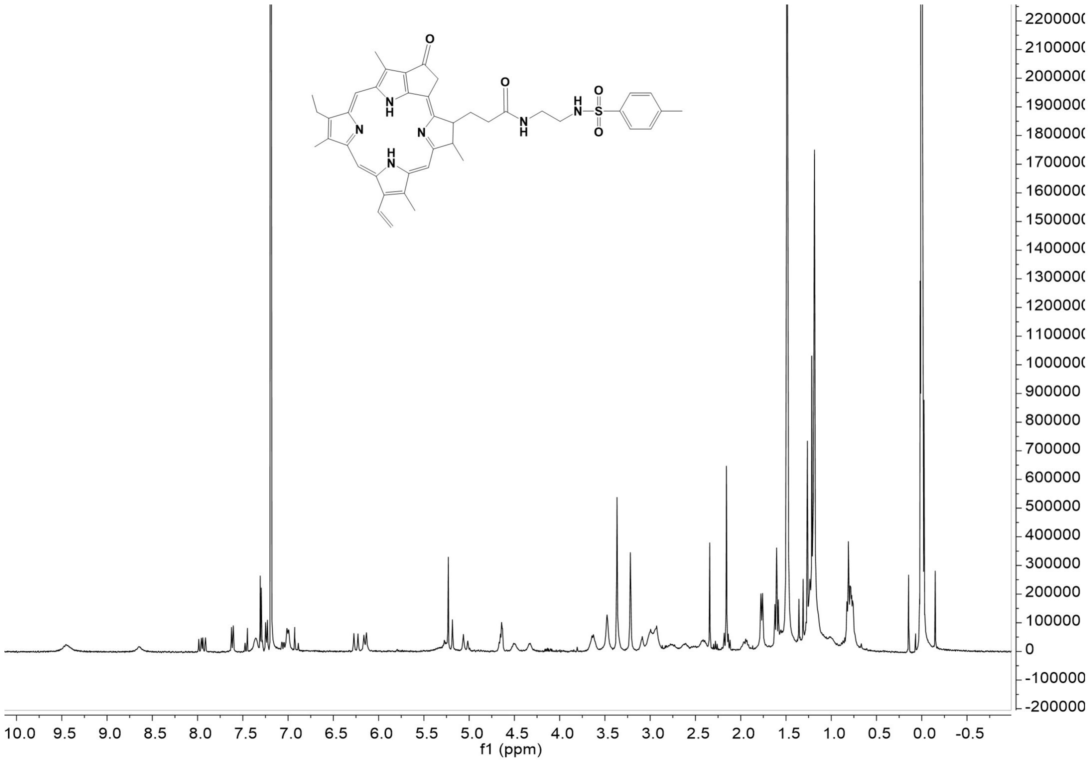

Figure S3. ^{1}H NMR spectrum of TPPa. Chloroform-d was used as solvent.

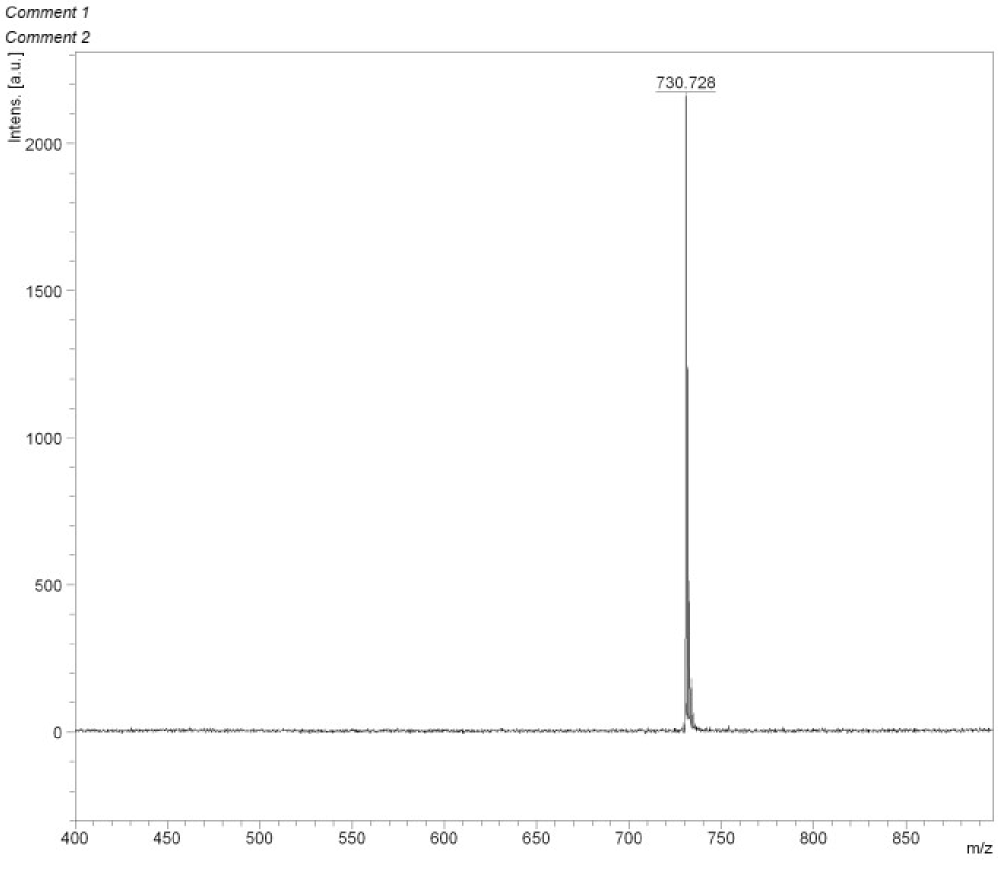

Figure S4. MALDI-TOF-MS of TPPa.

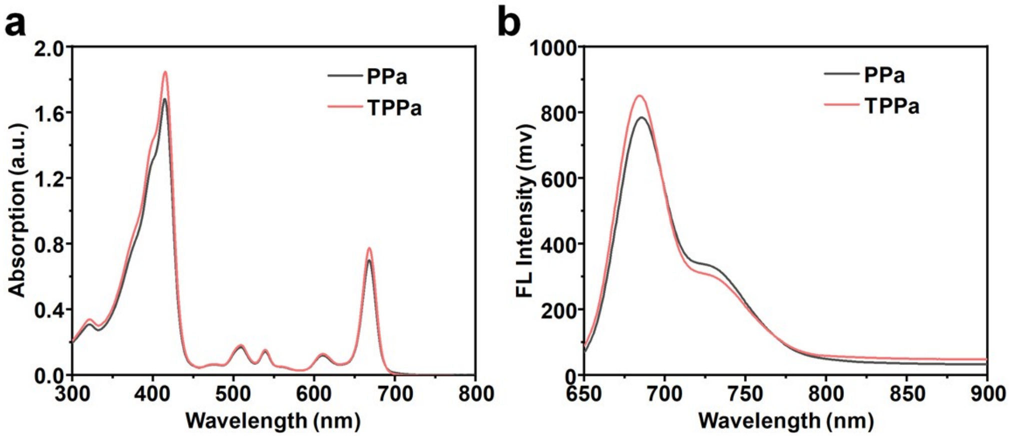

Figure S5. Absorption (a) and emission (b) spectra of PPa and TPPa in THF.

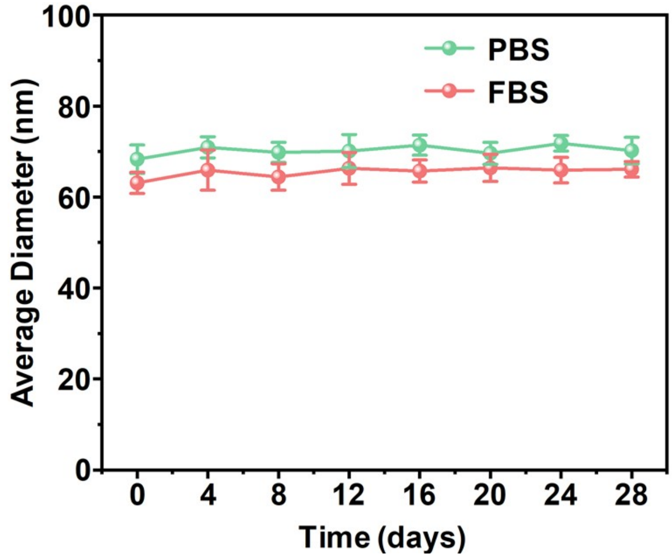

Figure S6. Average diameter changes of TPPa-Y NPs under different storage times.

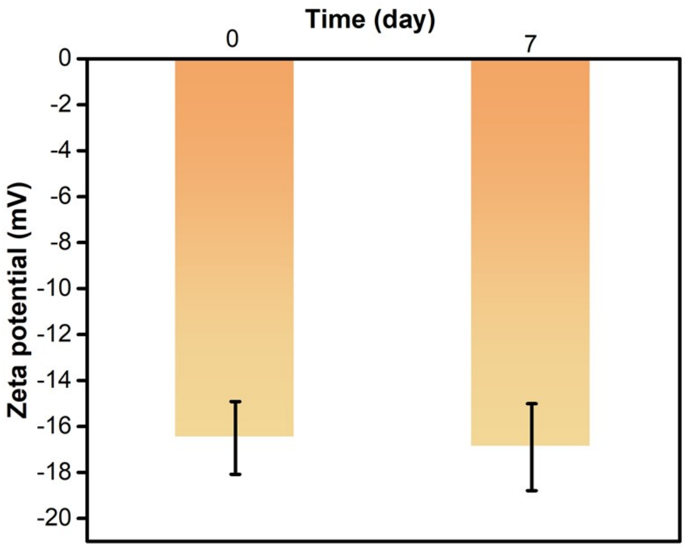

Figure S7. Zeta potential of TPPa-Y NPs after storage for 0 and 7 days.

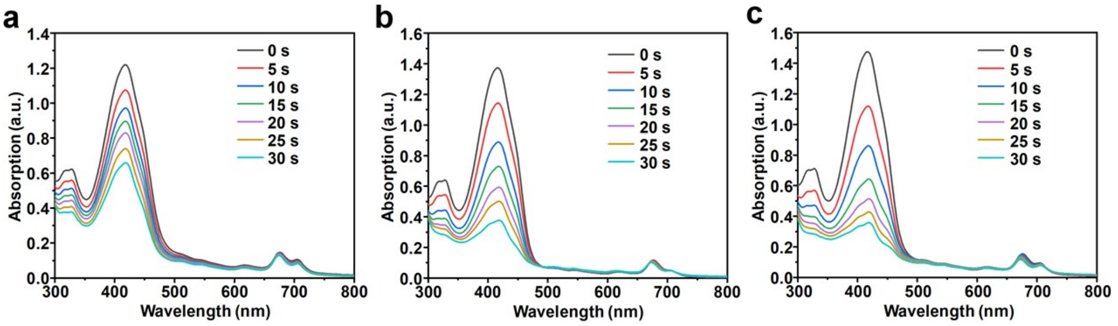

Figure S8. Absorption spectra changes of TPPa-Y NPs-incubated DPBF with 0.1 (a), 0.2 (b), and 0.3 (c) W/cm^2 635 nm laser irradiation for different times.

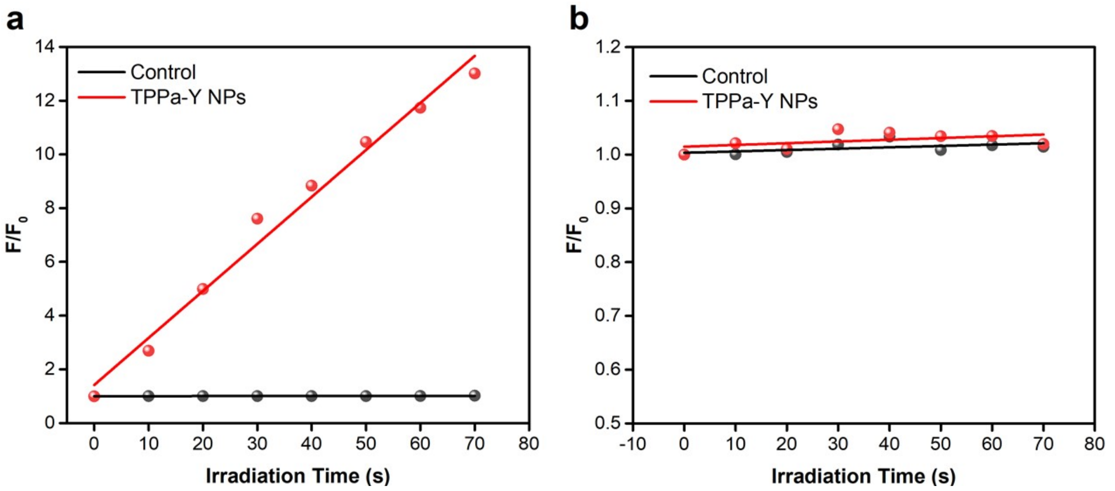

Figure S9. Fluorescence enhancement (F/F_0) of SOSG (a) or DHR-123 (b) in the absence or presence of TPPa-Y NPs as a function of 635 nm light irradiation time.

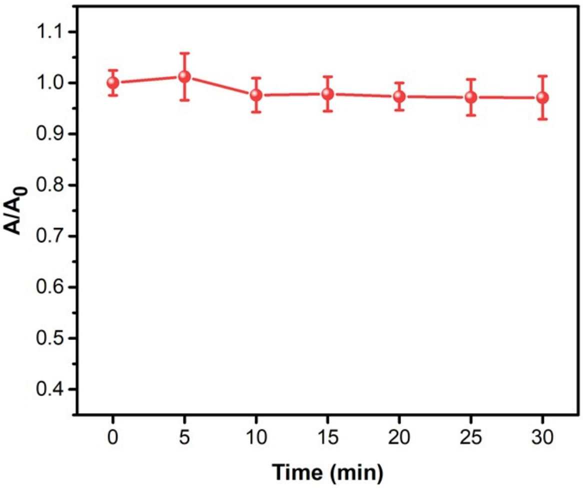

Figure S10. Normalized absorption changes of TPPa-Y NPs in the presence of H_2O_2

and BSA under 635 nm laser irradiation over time.

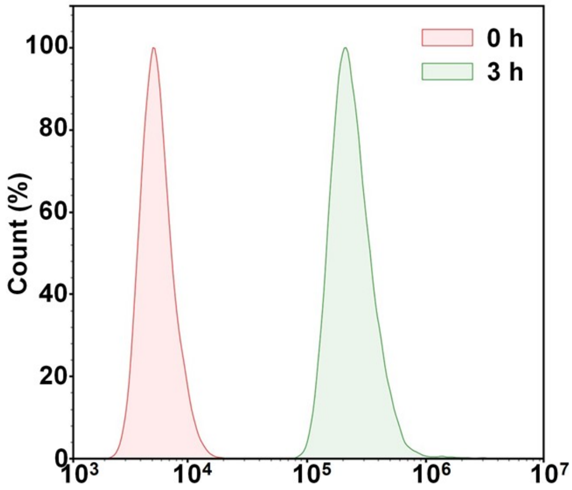

Figure S11. Flow cytometry analysis of fluorescence intensity of 4T1 cells incubated with TPPa-Y NPs for 3 h.

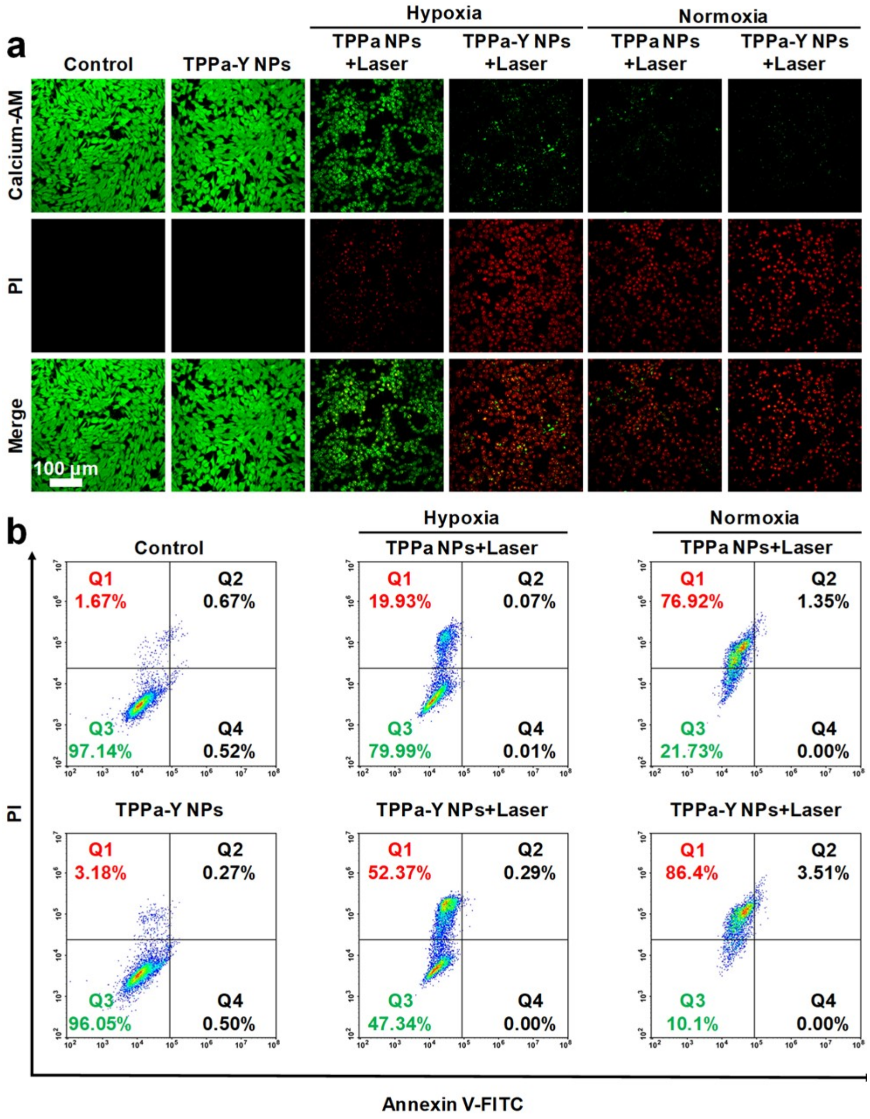

Figure S12. (a) Live/Dead assay of 4T1 cells under different treatment. (b) Representative flow cytometry plots of 4T1 cells under different treatments.

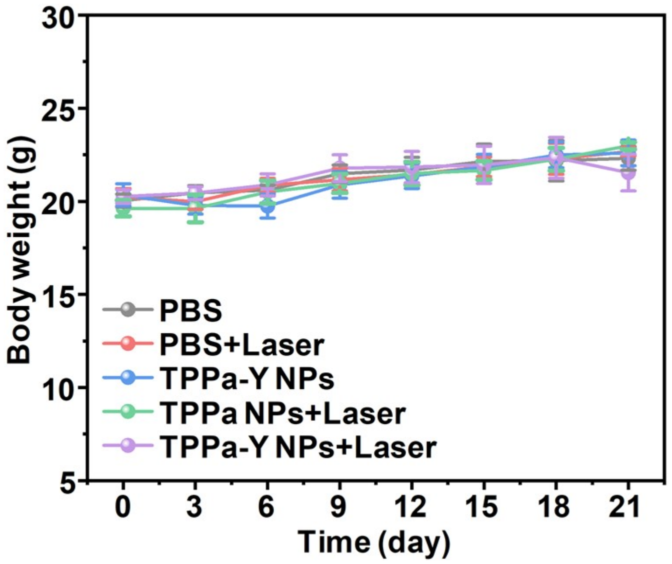

Figure S13. Average body weight of mice in each group during treatment.

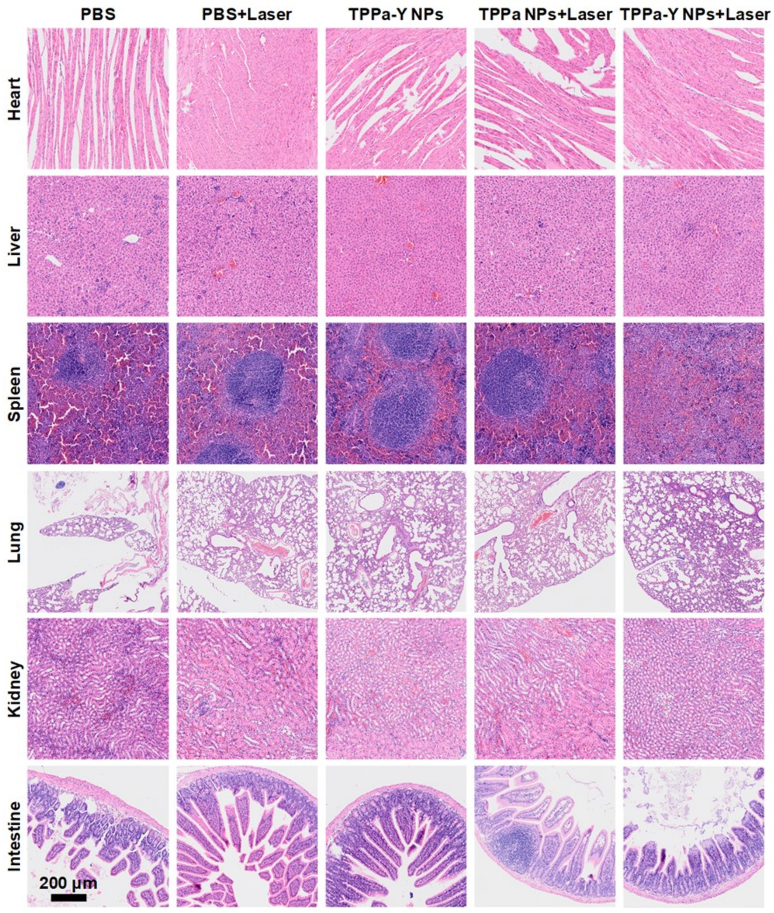

Figure S14. H&E staining of major organs from mice in each group.

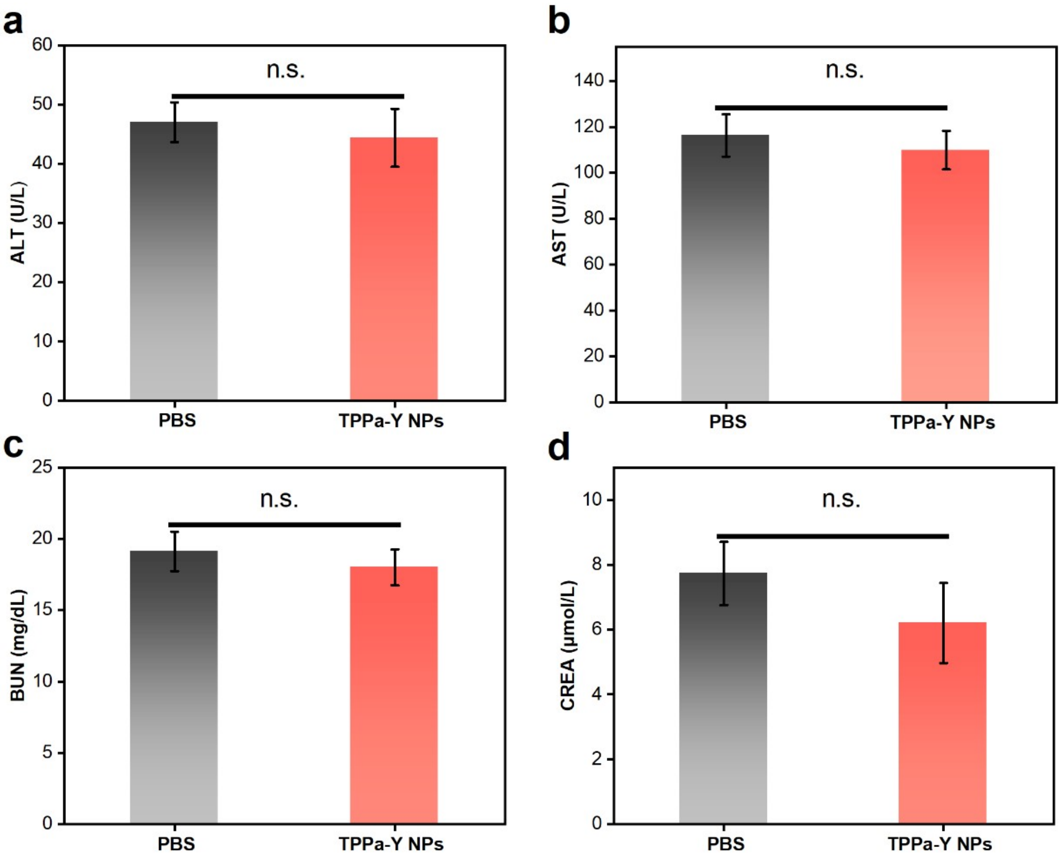

Figure S15. Biochemical analysis of ALT (a), AST (b), BUN (c) and CREA (d) in blood collected from mice after different treatments. The error bars represent standard deviations of five different measurements (n = 5). n.s.: not significant.

2. Experimental section

2.1 Materials and instruments

All chemicals in the experiments were received from Sigma-Aldrich and used without further purification unless otherwise mentioned. Proton nuclear magnetic resonance (^{1}H NMR) was conducted on a Bruker Ultra Shield Plus 400 MHz Spectrometer. $CDCl_3$ and Tetrahydrofuran-d8 were used as the solvents. The matrix-assisted laser desorption/ionization time-of-flight (MALDI-TOF) mass spectra were measured by a Bruker Autoflex TOF/TOF spectrometer. Transmission electron microscopy (TEM) measurements were carried out using a JEOL JEM-2100 transmission electron microscope operating at an acceleration voltage of 100.0 kV.

Dynamic light scattering (DLS) was obtained with a Brookhaven system. The UV-visible absorption spectra were obtained on a Shimadzu UV-3600 UV-VIS-NIR spectrophotometer. NIR fluorescence spectra were measured with a Shimadzu RF-6000 fluorescence spectrophotometer. Confocal fluorescence images were captured on a LSM880 confocal laser scanning microscope (Carl Zeiss, Germany). Flow cytometry analysis were conducted on a Flow Sight Imaging Flow Cytometer (Merck Millipore, Darmstadt, Germany). In vivo fluorescence images were recorded with PerkinElmer IVIS Lumina K, and fluorescence intensity was quantified by the region-of-interest measurement using Living image software (PerkinElmer). All the mice experiments were carried out in accordance with the guidelines of the Laboratory Animal Center of Jiangsu KeyGEN Biotech Corp., Ltd and approved by the Animal Ethics Committee of Simcere BioTech Corp., Ltd.

2.2 Synthesis of Compound 1

4-(Hydroxymethyl) phenylboronic acid pinacol ester (3 g, 12.8 mmol) and methacryloyl chloride (1.5 mL, 15.5 mmol) were dissolved in 20 mL of DCM, and then DMAP (2.4 g, 19.6 mmol) was slowly added to the mixture and stirred at room temperature for 24 h. After that, DCM was removed by distillation under reduced pressure, and the product was further isolated by column chromatography. purification, the product was purified by using petroleum ether (PE) and DCM (PE:DCM = 1:2) as eluent to obtain compound 1 (3.2 g, 82.8%).^{1}H NMR (400 MHz, Chloroform-d) δ: 7.86 – 7.82 (m, 2H), 7.42 – 7.38 (m, 2H), 6.18 (dd, J = 1.7, 1.0 Hz, 1H), 5.61 (p, J = 1.6 Hz, 1H), 5.23 (s, 2H), 1.99 (t, J = 1.3 Hz, 3H), 1.37 (s, 12H).

2.3 Synthesis of PEG-PMPAP

Compound 1 (150 mg, 1 mmol), PEG-RAFT (1 g, 0.1 mmol), and AIBN (2 mg) were added into a 20 mL polymerization tube and 5 mL of anhydrous THF was added to dissolve the solids. The reaction was carried out at 70 °C for 24 h under nitrogen atmosphere. After that, a large amount of light pink solid was precipitated by the addition of 100 mL of anhydrous diethyl ether, and the solid was washed with anhydrous diethyl ether three times. The product PEG-PMPAP (230 mg) was obtained after filtration under reduced pressure and followed by overnight drying in a vacuum

drying oven, and the degree of polymerization was verified by NMR to be about 11. ^{1}H NMR (400 MHz, THF-d8) δ: 7.75 (s, 2H), 7.32 (s, 2H), 4.87 (s, 2H), 3.46 (d, 44H), 1.27 (s, 12H).

2.4 Synthesis of TPPa

PPa (50 mg, 0.1 mmol), N-(2-aminoethyl)-4-methylbenzenesulfonamide (30 mg, 0.15 mmol), HATU (71 mg, 0.19 mmol), HOBT (25 mg, 0.19 mmol), and DIPEA (48 mg, 0.37 mmol) were dissolved in 5 mL of DMF and the reaction was carried out at room temperature for 24 h. After that, the solvent was removed by distillation under reduced pressure, and 10 mL of DCM was added to dissolve the mixture, which was extracted with saturated saline three times and dried with anhydrous Na_2SO_4. The product TPPa (36 mg, 53%) was further isolated and purified by column chromatography using DCM and MeOH (DCM:MeOH = 30:1) as eluent. ^{1}H NMR (400 MHz, Chloroform-*d*) *δ*: 9.45 (s, 1H), 8.65 (s, 1H), 7.95 (dd, J = 17.8, 11.6 Hz, 1H), 7.62 (d, J = 8.1 Hz, 1H), 7.43 – 7.26 (m, 2H), 7.00 (d, J = 7.9 Hz, 2H), 6.25 (d, J = 17.8 Hz, 1H), 6.15 (d, J = 11.5 Hz, 1H), 4.65 (d, J = 6.1 Hz, 1H), 4.50 (s, 1H), 4.33 (s, 1H), 3.66 – 3.60 (m, 1H), 3.48 (s, 1H), 3.37 (s, 2H), 3.22 (s, 1H), 3.09 (s, 1H), 2.34 (s, 1H), 2.16 (s, 2H), 1.99 – 1.91 (m, 1H), 1.77 (d, J = 7.3 Hz, 2H), 1.60 (t, J = 7.6 Hz, 2H), 1.31 – 1.12 (m, 15H), 0.83 – 0.78 (m, 3H). MS (MALDI-TOF, m/z) Calcd for $C_{42}H_{46}N_6O_4S$, 730.330; Found: 730.728.

2.5 Preparation of TPPa-Y NPs and TPPa NPs

TPPa-Y NPs were prepared by a nanoprecipitation method. TPPa (1 mg), YC-1 (0.5 mg), and PEG-PMPAP (20 mg) were dissolved into 1 mL of THF, and the mixed solution was rapidly injected into 10 mL of deionized water under vigorous sonication for 3 min. The nanoparticle solution was blown out of the THF under a gentle stream of nitrogen, and then filtered by 0.22 μm aqueous filtration membrane to get a dark-green and transparent solution and concentrated by ultracentrifugation and stored in a refrigerator at 4 °C. TPPa NPs were prepared via similar procedure without adding YC-1.

2.6 Measurement of singlet oxygen generation

Singlet oxygen generation of TPPa-Y NPs under 635 nm laser irradiation was

measured based on monitoring the absorption loss of 1,3-diphenylisobenzofuran (DPBF). DPBF were added into TPPa-Y solution (10 μg mL^{-1}) to a final concentration of 5 μg mL^{-1}. The obtained solution was irradiated under 635 nm laser with different power (0.25 W cm^{-2}, 0.5 W cm^{-2}, 0.75 W cm^{-2}), and the absorbance at 414 nm was monitored.

2.8 Drug release monitoring

The drug release profile of TPPa-Y NPs was tested under H_2O_2 concentrations of 0, 1, 5 and 10 mM, respectively. TPPa-Y NPs solutions (20 μg/mL, pH = 7.4) were divided into four groups and each group had 1 mL of solution. The solution was placed in a dialysis bag and sealed, then immersed into 5 mL of PBS solution (pH = 7.4) with different concentrations of H_2O_2. The samples were placed in a thermostatic incubator at 37 °C and 200 rpm. At time points of 2 h, 4 h, 6 h, 8 h, 10 h, 12 h, 24 h, 36 h, and 48 h, the PBS solutions were collected and another 5 mL fresh PBS solution was added, and the released drug in the collected PBS solution was determined by high performance liquid chromatography (HPLC).

2.9 Cellular uptake assays and targeting the endoplasmic reticulum

4T1 cells and NIH 3T3 cells were purchased from Jiangsu KeyGEN Biotech Corp. Ltd. 4T1 cells or NIH 3T3 cells were cultured in Dulbecco's Modified Eagle Medium (DMEM) with 10% fetal bovine serum (FBS) and antibiotics (10 mg mL^{-1} of streptomycin and 10 U mL^{-1} of penicillin) in an atmosphere of 5% carbon dioxide and 95% humidified air at 37 °C. Fluorescein isothiocyanate (FITC) was doped into TPPa-Y NPs using a nano-precipitation method similar to that described in 2.5 to give them green fluorescence for flow cytometric uptake experiments. 4T1 cells were inoculated into 12-well cell culture plates (2×10^5 cells per well), FITC-loaded TPPa-Y NPs (20 μg/mL) were added, and after incubation for 0, 3, 6, and 9 h, respectively, they were washed twice with fresh PBS as well as treated with EDTA-free trypsin, collected into 1 mL centrifuge tubes and centrifuged, and the cells were re-dispersed and mixed by adding 0.2 mL of PBS, which were analyzed by flow cytometry and the fluorescence signal was detected by flow cytometry.

CLSM imaging was further performed to confirm the following subcellular localization

of internalized TPPa-Y NPs. Cells were inoculated into confocal dishes, TPPa NPs (20 μg/mL) and PPa NPs (20 μg/mL, unmodified PPa) were added respectively, and the cells were washed by the addition of fresh DMEM after incubation for 12 h. Subsequently, the cells were stained for co-localization with commercial endoplasmic reticulum-targeting dye (ER-Tracker Green) for 15 min in an incubator, and the cells were stained with PBS to wash the cells three times, and 2 mL of DMEM incomplete medium was added and observed under a confocal microscope. The green channel is ER-Tracker Green and the red channel is TPPa.

2.10 In vitro intracellular HIF-1α inhibition assay.

In order to verify the inhibitory effect of nanoparticles on the expression of HIF-1α in 4T1 cells, this experiment was carried out under anoxic conditions. For this purpose, we set up a total of three sets of experiments: (1) PBS; (2) TPPa NPs; and (3) TPPa-Y NPs. 4T1 cells (~5×10^4) were inoculated into cell culture dishes and allowed to grow for 24 h. After 8 h of treatment in each of the above sets, the cells were stained with HIF-1α Antibody antibody at 4°C overnight, and then incubated at room temperature with Alexa Fluor 488-labeled goat anti-rabbit IgG secondary antibody was incubated for 1 h. Confocal imaging was performed after DAPI staining.

2.11. Cytotoxicity Assay (MTT).

The toxicity of TPPa-Y NPs to cells was evaluated by tetramethyl azole blue (MTT) assay. The procedure was as follows: 4T1 cells or NIH 3T3 cells were inoculated into 96-well plates (10,000 cells per well, 100 μL of medium) and cultured in an incubator for 24 h. TPPa NPs and TPPa-Y NPs were diluted to different concentrations (0, 2, 4, 8, 16, 32, and 64 μg/mL) with fresh medium and added to the above 96-well plates, and then cultured together with the 4T1 cells for 12 hours at 37 °C in a humidified incubator (normoxic environment) with 5% CO_2 or in a hypoxic bag. The cells were then placed in a humidified incubator (normoxic environment) or hypoxic bag, in which NIH 3T3 cells were incubated in a normoxic environment with TPPa-Y NPs material only. The cells were incubated at 37°C, 5% CO_2 for 12 hours. Each well was irradiated with a 635 nm laser (0.1 W/cm^2) for 1 min, no laser irradiation was required for NIH 3T3 cells. Subsequently, the original medium was replaced with

fresh medium containing MTT (20 μL, 5 mg/mL), and the cells were placed in an incubator to continue incubation for 4 h. The top layer of medium was then aspirated, and 100 μL of DMSO was added to each well to dissolve the purple solids at the bottom. DMSO was added to each well to dissolve the purple solid at the bottom, and the absorbance at 450 nm was detected by an enzyme meter after shaking well. Cell viability was calculated by the ratio of the absorbance value of cells cultured with TPPa NPs and TPPa-Y NPs to that of cells cultured with normal medium.

2.12. Intracellular ROS detection.

4T1 cells were inoculated in confocal culture dishes for 24 h. TPPa NPs and TPPa-Y NPs (20 μg/mL) diluted with DMEM incomplete medium were then added to the dishes, which were incubated with the cells in a humidified incubator (normoxic environment) with 5% CO_2 and a depleted-oxygenation bag for 12 h. Subsequently, the DCHF-DA was diluted 1000 fold in PBS buffer. Then, the upper layer of culture fluid from the confocal dish was aspirated and 1 mL of newly conFig. d DCFH-DA solution was added, and incubated in the incubator for 15 min, followed by washing twice with PBS and adding 2 mL of DMEM incomplete medium. Confocal images of each group were acquired after irradiation with a 635 nm laser (0.1 W/cm^2) for 30 s.

2.13. Live/Dead assay.

4T1 cells (~5 × 10^4) were seeded into a cell culture dish and allowed to grow for 24 h, and the cells were randomly divided into six groups, which were (1) control, (2) TPPa-Y NPs, (3) TPPa NPs +Laser (Hypoxia), (4) TPPa -Y NPs +Laser (Hypoxia), (5) TPPa NPs +Laser (Normoxia) , (6) TPPa-Y NPs +Laser (Normoxia). For dead/live staining, 4T1 cells were incubated with TPPa NPs/ TPPa -Y NPs (64 μg mL^{-1}) for 24 h. 4T1 cells without TPPa NPs/ TPPa -Y NPs were used as control. Then, the medium was removed and washed with cold PBS buffer three times. Fresh DMEM medium (0.5 mL) was added to dishes, and 4T1 cells in each dish were treated with laser irradiation for 1 min (635 nm, 0.25 W cm^{-2}). After irradiation, the medium was removed carefully, and 1 μM Calcein AM and 1 μM PI were added into each dish and incubated for 30 min. The medium was then removed, washed with PBS three times, and the fluorescence images of Calcein AM and PI were obtained on an LSM880 confocal laser

scanning microscope.

2.14. Apoptosis evaluation by flow cytometry.

4T1 cells (~5 × 10^5) were seeded into 6-well plates and incubated at 37 °C for 24 h, and the cells were randomly divided into four groups, which were (1) control, (2) TPPa-Y NPs, (3) TPPa NPs +Laser (Hypoxia), (4) TPPa -Y NPs +Laser (Hypoxia), (5) TPPa NPs +Laser (Normoxia), (6) TPPa-Y NPs +Laser (Normoxia). After removing the medium, TPPa NPs (64 μg mL^{-1}) were added to 4T1 cells in fresh DMEM medium (1.5 mL) for groups 3 and 5, and TPPa -Y NPs (64 μg mL^{-1}) were added to 4T1 cells in group 2, 4, and 6, then the cells were incubated for 12 hours at 37 °C. Blank 4T1 cells were set as control. After incubation, the medium was removed, and washed with PBS (0.01 M, pH = 7.4), and fresh culture medium was then added. The cells were then treated with or without 635 nm laser irradiation (0.25 W cm^{-2}) for 1 min. After removal of the medium, the cells were trypsinized, collected, resuspended in 0.2 mL PBS, and stained with Annexin V-FITC/PI. After staining, the apoptotic cell population was analyzed by Flow Sight Imaging Flow Cytometer. For each group, 10000 live cells were analyzed.

2.15. Tumor mouse model.

All the mice experiments were carried out in accordance with the guidelines of the Laboratory Animal Center of Jiangsu KeyGEN Biotech Corp., Ltd and approved by the Animal Ethics Committee of Simcere BioTech Corp., Ltd. The approval number was IACUC-004. 1 million 4T1 cells were inoculated at the flank region of the right arm of balb/c mice to establish the tumor model. The volume of tumor was calculated as follows:

$$V = Dd^2/2$$

V represents the volume of tumor, D and d represent maximum and minimum diameter of tumor, respectively.

2.16. In Vivo Fluorescence Imaging.

Female 4T1 tumor bearing BALB/c nude mice were randomly divided into two groups which injected intravenously with TPPa -Y NPs (100 μL, 400 μg mL^{-1}). Whole-body fluorescence images were then acquired with PerkinElmer IVIS Lumina K

(excitation wavelength 640 nm; emission wavelength 710 nm long pass) at 0, 0.5, 6, 12, 24, 48, 72 h post-injection. In an in vitro biodistribution study, mice were executed 72 h after injection, then tumors and major organs (heart, liver, spleen, lung, kidney, and intestine) were collected. Fluorescence images of these organs were acquired and the fluorescence intensity was quantified to evaluate the biodistribution of these two NPs. The fluorescence intensity of the tumor was quantified by region of interest (ROI). Measurements were performed using in vivo image software (PerkinElmer, MA, USA). Each experiment was performed in three mice.

2.17. In Vivo Anticancer Study.

4T1 tumor-bearing balb/c mice were randomly divided into five groups, each consisting of five mice. For the groups without laser irradiation, mice were intravenously injected with PBS (100 μL) or TPPa-Y NPs (100 μL, 400 μg/mL). For the laser irradiation groups, the mice's tumors were irradiated with a 635 nm laser (0.3 W/cm^2) for 10 minutes following intravenous injection of PBS (100 μL), TPPa NPs (100 μL, 400 μg/mL), or TPPa-Y NPs (100 μL, 400 μg/mL) for 12 hours. Tumor volume and body weight of mice in each group were measured every other day for 21 days. For histological studies, mice were sacrificed at day 21, and tumor tissues along with major organs were collected and stained with H&E.

2.18 Data analysis

Fluorescence intensity in images were calculated by region of interest (ROI) analysis using IVIS system. Results were shown as mean ± SD unless otherwise mentioned. Statistical analysis of results was carried out by one-way analysis of variance (ANOVA) with a Tukey post-hoc test and two-tailed Student's t-test using GraphPad Prism version 8.0.2 (GraphPad Software, Inc., CA, USA). For statistical analysis, *$p < 0.05$, **$p < 0.01$, ***$p < 0.001$ were regarded as statistically significant.
